# **Health and Social Problems of Rhinoplasty in Iran**

**Published:** 2016-01

**Authors:** Mohammad Hosein Kalantar Motamedi, Ali Ebrahimi, Amin Shams, Nasrin Nejadsarvari

**Affiliations:** 1 Department of Plastic Surgery, Trauma Research Center, Baqiyatallah University of Medical Sciences, Tehran, Iran;; 2 Department of Medical Ethics, Tehran University of Medical Sciences, Tehran, Iran

**Keywords:** Iran, Rhinoplasty, Health

**DEAR EDITOR**

Rhinoplasty is the most popular cosmetic surgical procedure in Iran. Akbari Sari et al. in 2011 reported 180 cases of rhinoplasty per 100,000 populations (a total of 134,766 surgeries per year).^1^ This is considered as one of the world’s highest rates of rhinoplasty.^2^ Because of the Islamic dress code for women practiced in Iran, which leaves only their faces exposed, the nose is very prominent; thus, rhinoplasty has become the most popular plastic procedure in the country.^3^ More recently young men also are pursuing rhinoplasty and some of them for the 2^nd^ or 3^rd^ time.

Evaluation of personality traits and expectations of these patients are necessary before operation, because many of them have body dysmorphic disorder and mental abnormality.^3^^,^^4^ These candidates, may be complicated and dissatisfied after surgery. Nowadays in Iran, nose job is not just a cosmetic surgery to correct aesthetic defects; it has become a part of a fashion trend. Many consider a nose job a luxury to show their financial power and make girls marriageable; others wish to boost their self-confidence. 

Even during the recent economic crisis, some take to heavy bank loans to afford a rhinoplasty.

Although Iran is named the rhinoplasty capital of the world,^5^^-^^8^ most of the rhinoplasty procedures are done by unlicensed practitioners that cause some health and social problems. Iran’s medical council does not interfere with these operations that were done by non-qualified physicians unless malpractices suits are filed and increasing rates of post-rhinoplasty complications are being seen. 

Although Iranian society of plastic surgeons have complains regarding to unethical intervention of non-qualified physicians in cosmetic procedures, but because of the lack of clear rules as well as financial incentives and other problems, other medical groups have involved in aesthetic surgery without any specialized training. To resolve this social dilemma of malpractice, Iran’s Medical Council should reconsider its approach to this serious medical problems and prevent from this medical errors. Today rhinoplasty is not only a simple cosmetic or mental problem, but also as days go by, is becoming an epidemic in Iran, wasting a lot of financial and human resources and seems hard to stop. So after the wave of rhinoplasty operations, now Iran has to deal with the sunami of post-surgery complications than can be a health and ethical issue that should be considered by authorities in Iran’s Medical Council.

## CONFLICT OF INTEREST

The authors declare no conflict of interest.
